# *Notes from the Field:* Pediatric Intracranial Infections — Clark County, Nevada, January–December 2022

**DOI:** 10.15585/mmwr.mm7222a4

**Published:** 2023-06-02

**Authors:** Jessica A. Penney, Ying Zhang, Taryn Bragg, Rachel Bryant, Cassius Lockett

**Affiliations:** ^1^Epidemic Intelligence Service, CDC; ^2^Southern Nevada Health District, Las Vegas, Nevada; ^3^Intermountain Primary Children’s Hospital, Las Vegas, Nevada; ^4^Sunrise Children’s Hospital, Las Vegas, Nevada.

In October 2022, the Southern Nevada Health District (SNHD) was notified of a higher-than-expected number of pediatric patients hospitalized with intracranial abscesses; similar concerns were previously reported nationally (*1*,*2*). This rare infection is associated with significant morbidity (*3*,*4*). When SNHD received the report in October 2022, 14 cases had been diagnosed in the largest pediatric hospital in southern Nevada. SNHD investigated the reported increase to confirm that a cluster had been detected, identify common risk factors for infection, report findings to the community, and recommend measures to prevent future cases.

The observed and expected number of cases were compared to confirm and describe the cluster. Historical median quarterly case numbers with IQRs were obtained from discharge data from all hospitals in Clark County, Nevada during January 2015–December 2021. Persons with primary, secondary, or tertiary discharge diagnoses of intracranial abscess and granuloma (*International Classification of Diseases, Tenth Revision, Clinical Modification* [ICD-10-CM] code G06.0) or extradural and subdural abscess, unspecified (ICD-10-CM code G06.2) during January 2015–December 2022 among persons aged ≤18 years were identified as cases. Because hospital discharge data from the final quarter of 2022 were not available at the time of investigation, cases in 2022 were primarily identified through provider reporting and confirmed by discharge data, if available; for these data, a case was defined as diagnosis of an intraparenchymal abscess, subdural abscess or empyema, epidural abscess or empyema, or evidence of other intracranial extension observed on brain imaging in a person aged ≤18 years without a previous neurosurgical procedure or history of significant head trauma. Detailed medical chart abstraction and semistructured telephone interviews with families affected during 2022 were conducted to ascertain clinical course, risk factors, and exposures. This activity was reviewed by CDC and was conducted consistent with applicable federal law and CDC policy.*

During 2015–2021 overall, a median of one case per quarter (IQR = 0–2.0) was identified in Clark County. However, during the period preceding the COVID-19 pandemic (2015–2019), the quarterly median was 0.5 cases (IQR = 0–2.0), and during the first 2 years of the pandemic (2020–2021), the median number of quarterly cases reported was 1.5 (IQR = 0–2.5). During 2022, 18 cases were identified (median = five per quarter; IQR = 3.5– 6.0); all occurred after February 2022 (Figure).

**FIGURE:**
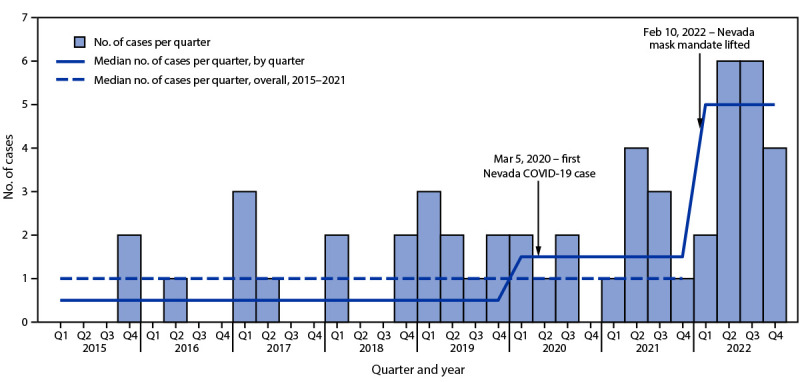
Number of cases of pediatric intracranial infections and median number of infections per quarter — Clark County, Nevada, 2015–2022 Abbreviation: Q = quarter.

Review of medical charts of the 18 cases reported in 2022 found that the median patient age was 12 years (range = 4–15 years) and that all but four cases occurred in males. Children and adolescents were hospitalized for a median of 15 days (range = 9–76 days), and 15 patients required craniotomy for abscess drainage. Sinusitis was diagnosed in 14 patients and mastoiditis in four. No patients received a positive test result for SARS-CoV-2 on admission. No associated deaths were reported.

Telephone interviews were conducted with 14 caregivers as a proxy for the affected child or adolescent, nine of whom reported that the child had cold symptoms, including rhinorrhea, before hospitalization; seven experienced other symptoms, including headache (three), headache with fever (three), and mild head injuries (two).^†^ Eleven caregivers sought care for their child before hospitalization, most often at an emergency department (seven). The median interval from symptom onset to hospitalization was 7 days (range = 2–14 days). Nine interviewees reported that the child had been swimming during the 4 weeks preceding hospitalization, but not at the same pool locations. Five interviewees reported cessation of masking practices after the COVID-19 mask mandate was lifted,^§^ including three who reported cold symptoms experienced by the affected child before hospitalization.

A 2022 investigation of possible increased incidence of pediatric intracranial abscesses identified a higher number of cases in 2022 compared with that reported in 2021 (*2*). Contributing to this increase was a period of elevated cases beginning in mid-2021, which followed a period of consistently low case counts after the onset of the pandemic (*2*). This pattern was also observed in the current investigation. Although this investigation did not identify unexpected risk factors for intracranial abscesses, the substantial increase in cases after the mask mandate in Nevada was lifted might be partially attributable to changes in respiratory pathogen transmission. SNHD released a health advisory notice to pediatric health care providers detailing the investigation findings; surveillance will be continued through 2023 to better monitor trends in incidence of pediatric intracranial infections.

## Footnotes

* 45 C.F.R. part 46.102(l)(2), 21 C.F.R. part 56; 42 U.S.C. Sect. 241(d); 5 U.S.C. Sect. 552a; 44 U.S.C. Sect. 3501 et seq.

† Reported symptoms and injuries are not mutually exclusive.

§ Nevada mask mandate was in effect during July 19, 2021–February 10, 2022, and required use of face masks in indoor public areas including schools.
